# 8q24 clear cell renal cell carcinoma germline variant is associated with *VHL* mutation status and clinical aggressiveness

**DOI:** 10.1186/s12894-020-00745-9

**Published:** 2020-10-29

**Authors:** Jeanette E. Eckel-Passow, Huihuang Yan, Matthew L. Kosel, Daniel Serie, Paul A. Decker, Robert B. Jenkins, Brian Costello, Bradley Leibovich, Thai H. Ho, Alexander Parker

**Affiliations:** 1 Division of Biomedical Statistics and Informatics, Mayo Clinic, 200 First Street SW, Rochester, MN 55905 USA; 2 Department of Health Sciences Research, Mayo Clinic, Jacksonville, FL USA; 3 Department of Laboratory Medicine and Pathology, Mayo Clinic, Rochester, MN USA; 4 Department of Urology, Mayo Clinic, Rochester, MN USA; 5 Division of Hematology and Medical Oncology, Mayo Clinic, Scottsdale, AZ USA

**Keywords:** Kidney, GWAS, Subtype, Hi-C

## Abstract

**Background:**

The four most commonly-mutated genes in clear cell renal cell carcinoma (ccRCC) tumors are *BAP1*, *PBRM1*, *SETD2* and *VHL*. And, there are currently 14 known RCC germline variants that have been reproducibly shown to be associated with RCC risk. However, the association of germline genetics with tumor genetics and clinical aggressiveness are unknown.

**Methods:**

We analyzed 420 ccRCC patients from The Cancer Genome Atlas. Molecular subtype was determined based on acquired mutations in *BAP1*, *PBRM1*, *SETD2* and *VHL*. Aggressive subtype was defined clinically using Mayo SSIGN score and molecularly using the ccA/ccB gene expression subtype. Publically-available Hi-C data were used to link germline risk variants with candidate target genes.

**Results:**

The 8q24 variant rs35252396 was significantly associated with *VHL* mutation status (OR = 1.6, *p* = 0.0037) and SSIGN score (OR = 1.9, *p* = 0.00094), after adjusting for multiple comparisons. We observed that, while some germline variants have interactions with nearby genes, some variants demonstrate long-range interactions with target genes.

**Conclusions:**

These data further demonstrate the link between rs35252396, *HIF* pathway and ccRCC clinical aggressiveness, providing a more comprehensive picture of how germline genetics and tumor genetics interact with respect to tumor development and progression.

## Background

The majority (> 90%) of kidney cancer is classified as renal cell carcinoma (RCC) and approximately 85% of RCCs are further classified as the clear cell subtype (ccRCC). The etiology of ccRCC has been extensively studied and smoking, obesity and hypertension are recognized environmental risk factors that increase the risk of developing ccRCC. Additionally, genome-wide association studies (GWAS) have to date identified 14 germline variants that are associated with risk of RCC [1–5]. The value of these germline genetic explorations notwithstanding, the functional impact of the germline variants associated with RCC and ccRCC specifically remains largely unknown. Furthermore, the association of germline genetics with tumor genetics and tumor aggressiveness are largely unknown. In some cancers investigators have reported that germline variants are associated with specific molecularly-defined tumor subtypes, and in some cases the association is large enough to suggest clinical relevance (e.g., rs55705857 has an odds ratio > 6 in *IDH*-mutated glioma) [6–8]. To date, similar analyses linking germline variants with tumor subtypes have not been performed for RCC or ccRCC specifically. The value of identifying associations of genetic variants with specific molecular subtypes of a tumor centers on the ability to provide evidence of the involvement of specific developmental pathways that can help inform the biology of ccRCC tumor evolution, progression and novel prevention efforts. Related to this, we now have access to a catalog of acquired tumor alterations that are commonly present in ccRCC [9, 10]. Motivated by the opportunity to combine data on germline genetics associated with ccRCC risk with specific acquired molecular alterations found in ccRCC tumor tissue, we advance the field by evaluating for the first time the association of known ccRCC germline variants with these acquired alterations in order to better understand ccRCC development and progression. Moreover, we also evaluated the association of these known germline variants with well-known and validated clinical measures of ccRCC aggressiveness. Finally, we leverage Hi-C data to identify candidate target genes for the 14 germline variants that are the focus of this investigation.

## Methods

### The Cancer Genome Atlas (TCGA) data

Raw genotyping data from Affymetrix 6.0 array were obtained from germline DNA for 420 ccRCC patients, as well as corresponding clinical and pathological data. Somatic mutations of the four most frequently-mutated genes in ccRCC (*BAP1*, *PBRM1*, *SETD2* and *VHL*) were obtained from TCGA [9]. The clear cell A and clear cell B (ccA/ccB) gene expression subtype classification has been reproducibly shown to be associated with outcome [11, 12] and was obtained from [9]. The Mayo SSIGN score has also been reproducibly shown to be associated with outcome [13–16]. The Mayo SSIGN score is derived from an additive model that contains tumor stage, tumor size, tumor grade and presence of necrosis, and was calculated as described previously [17].

### Statistical methods

Quality control was performed on the genotying data for the 420 ccRCC TCGA patients, including 95% call rate (zero germline variants failed), 95% sample call rate (zero samples failed), Hardy–Weinberg equilibrium (821 variants failed with *p* < 0.000001), minor allele frequency (MAF; 216,942 variants had MAF < 0.05), sex check (100% concordance) and population stratification (all Caucasian). Genotyping data were phased and imputed using the Michigan Imputation Server with the Haplotype Reference Consortium (release 1) as the reference population. The imputation quality for each of the 14 variants is provided in Additional file 1: Table S1. An additive logistic regression model was used to assess the association between each of the 14 variants and subtype, with genotype coded as having 0, 1, or 2 copies of the minor allele for observed data and dosage was modeled as continuous for imputed data. A general linear model was used to assess the association between age of diagnosis and each of the 14 variants. To account for multiple testing, *p* values < 0.004 were considered statistically significant (0.05/14 = 0.004).

### Hi-C analysis

Associations between known RCC germline variants and genes within two-to-five megabase (Mb) were evaluated using Hi-C data via the HUGIn web browser [18]. Additional genetic information, limited to what was available in HUGIn, was included such as frequently interacting regions (FIREs), topologically associating domain (TAD) boundary regions, and occupancy of histone marks H3K27ac, H3K4me1 and H3K4me3. Because RCC is thought to originate from the renal tubular epithelium, and mesodermal stem cells form the tubule of the kidney, analyses were performed using mesendoderm cells. Mesenchymal stem cells were also analyzed [19]; results were similar across the two cell lines. We also used Hi-C data from ccRCC cell line (*VHL *mutant) Caki2 (GSM2827127 and GSM2827128) [20], (*VHL* wild-type) embryonic kidney cell lines HEK293T (GSM1081530 and GSM1081531) and HEK293T RAD21cv that was treated with tobacco etch mosaic virus protease (GSM1081526 and GSM1081527) [21]. RAD21 is a core subunit of cohesin complex, which is known to play a role in mediating chromosomal loops. In RAD21cv (a RAD21-EGFP variant) cells, RAD21cv replaced endogenous RAD21 and was incorporated into the cohesin complex. For Caki2 and HEK293 data, reads were mapped with Bowtie 2 [22] and alignments from two replicates were combined. Chromatin interaction was identified with HOMER (https://homer.ucsd.edu/homer/interactions/) at 20-kb resolution, which takes into account the dependence of interaction frequency and linear distance along each chromosome. For each risk locus, we combined the virtual 4C plot generated by the HUGIn web browser with the interaction plot generated from internally-analyzed Hi-C data.

## Results

### TCGA ccRCC cohort

Table 1 describes the 420 TCGA ccRCC patients that were analyzed. Of the 376 patients with available whole exome sequencing data, 150 (40%) had a *VHL* mutation, 34 (9%) *BAP1* mutation, 119 (32%) *PBRM1* mutation and 48 (13%) *SETD2* mutation. We also subtyped patients according to disease aggressiveness using pathological indices defined by the Mayo SSIGN score [13] as well as molecularly according to the ccA/ccB gene expression subtype [11, 12]. Of the 355 patients that had available pathology data to calculate the Mayo SSIGN score, 81 (23%) were classified as aggressive (SSIGN score > 8). Using ccA/ccB to classify aggressiveness, of the 352 patients who had data, 168 (48%) were poor prognosis (ccB) subtype.

**Table 1:** Description of 420 TCGA ccRCC patients

	N (%)
Sex	
Female	137 (32.6%)
Male	283 (67.4%)
Max tumor size	
N	376
Mean (SD)	6.5 (3.5)
Median	5.5
Q1, Q3	4.0, 8.5
Range	(1.1–25.0)
Stage	
Missing	44
Stage I	176 (46.8%)
Stage II	135 (35.9%)
Stage IV	65 (17.3%)
Grade	
Missing	44
G1	7 (1.9%)
G2	156 (41.5%)
G3	152 (40.4%)
G4	60 (16.0%)
GX	1 (0.3%)
Percent necrosis	
Missing	64
0	189 (53.1%)
2	4 (1.1%)
3	2 (0.6%)
5	42 (11.8%)
8	1 (0.3%)
10	23 (6.5%)
15	13 (3.7%)
20	6 (1.7%)
25	4 (1.1%)
30	72 (20.2%)
SSIGN group	
Missing	65
Low risk (0–3)	159 (44.8%)
Intermediate risk (4–7)	115 (32.4%)
High risk (8+)	81 (22.8%)
BAP1 mutated	
Missing	44
No	342 (91.0%)
Yes	34 (9.0%)
PBRM1 mutated	
Missing	44
No	257 (68.4%)
Yes	119 (31.6%)
SETD2 mutated	
Missing	44
No	328 (87.2%)
Yes	48 (12.8%)
VHL mutated	
Missing	44
No	226 (60.1%)
Yes	150 (39.9%)
ccA/ccB expression subtype	
Missing	68
ccB	168 (47.7%)
ccA	184 (52.3%)

### Association of RCC germline variants with frequently mutated genes

Using a case-case design, we evaluated the association of each of the 14 RCC germline variants with known ccRCC acquired alterations in *BAP1*, *PBRM1*, *SETD2* and *VHL* (Table 2). We observed a statistically significant association after adjusting for multiple comparisons between the 8q24 variant rs35252396 and *VHL* mutation (OR = 1.60, *p* = 0.0037). While not significant after adjusting for multiple testing, we also observed a candidate association between *EPAS1* variant rs7579899 and *SETD2* mutation (OR = 1.87, *p* = 0.012) (Table 2).

**Table 2:** Association of known RCC germline variants with RCC subtypes. Subtypes were defined molecularly based on individual acquired alterations, by mRNA molecular subtype (ccA/ccB) or clinically (Mayo SSIGN score).

RS ID	Chrom	REF	ALT	OR.bap1	P.bap1	OR.pbrm1	P.pbrm1	OR.setd2	P.setd2	OR.vhl	P.vhl	OR.ccAccB	P.ccAccB	OR.SSIGNcat	P.SSIGNcat
rs4381241	1	T	C	0.92	0.74	0.92	0.59	0.94	0.77	0.92	0.59	0.95	0.72	1.10	0.61
rs7579899	2	A	G	0.95	0.85	1.02	0.92	1.87	**0.012**	0.99	0.93	1.12	0.45	0.95	0.79
rs12105918	2	T	C	2.05	0.05	0.66	0.16	0.58	0.23	1.36	0.23	0.86	0.57	0.84	0.58
rs67311347	3	G	A	1.29	0.33	1.00	0.98	1.04	0.88	1.17	0.33	1.02	0.90	0.89	0.57
rs10936602	3	T	C	1.22	0.49	0.77	0.18	0.73	0.26	1.11	0.54	1.15	0.43	0.96	0.8
rs2241261	8	C	T	0.96	0.88	1.03	0.84	0.89	0.60	1.26	0.12	1.07	0.66	0.84	0.32
rs35252396^a^	8	A	C	1.17	0.57	0.95	0.77	0.97	0.89	1.60	*0.0037*	0.99	0.96	1.92	*0.00094*
rs35252396^a^	8	C	G	1.17	0.57	0.95	0.77	0.97	0.89	1.60	*0.0037*	0.99	0.96	1.92	*0.00094*
rs11813268	10	C	T	1.15	0.64	0.85	0.42	0.54	0.06	0.88	0.50	1.02	0.93	1.14	0.54
rs7105934	11	G	A	0.32	0.27	0.96	0.92	0.46	0.29	0.72	0.40	0.56	0.13	0.66	0.41
rs1800057	11	C	G	1.68	0.44	1.64	0.29	0.47	0.39	0.92	0.86	1.34	0.53	0.66	0.51
rs74911261	11	G	A	2.21	0.25	1.13	0.82	0.31	0.31	0.49	0.21	1.29	0.63	0.76	0.68
rs718314	12	A	G	1.05	0.86	0.93	0.67	0.89	0.65	1.11	0.55	0.94	0.72	1.07	0.74
rs4765623	12	C	T	1.13	0.65	0.99	0.94	0.64	0.07	1.08	0.63	0.92	0.60	0.95	0.77
rs4903064	14	T	C	1.35	0.25	0.90	0.55	1.19	0.45	1.06	0.72	1.04	0.83	0.96	0.82

Chrom denotes chromosome location, OR denotes odds ratio and *p* value is from a logistic regression model. Cells highlighted in italics denote associations that pass our multiple testing threshold (*p* value < (0.05/14 = 0.004). Cells highlighted in bold denote candidate associations (0.004 < *p* value < 0.05)

^a^rs35252396 is tri-allelic

### Association of RCC germline variants with aggressive ccRCC

We observed a statistically significant association between the 8q24 variant rs35252396 and Mayo SSIGN score (OR = 1.92, *p* = 0.00094) (Table 2). However, we did not observe a statistically-significant association between the known germline variants and ccA/ccB gene expression subtype.

### Association of RCC germline variants with age at diagnosis

We did not observe a statistically-significant association between the known germline variants and age at diagnosis (Additional file 1: Table S2).

### Association of RCC germline variants with nearby genes

**Fig. 1:**
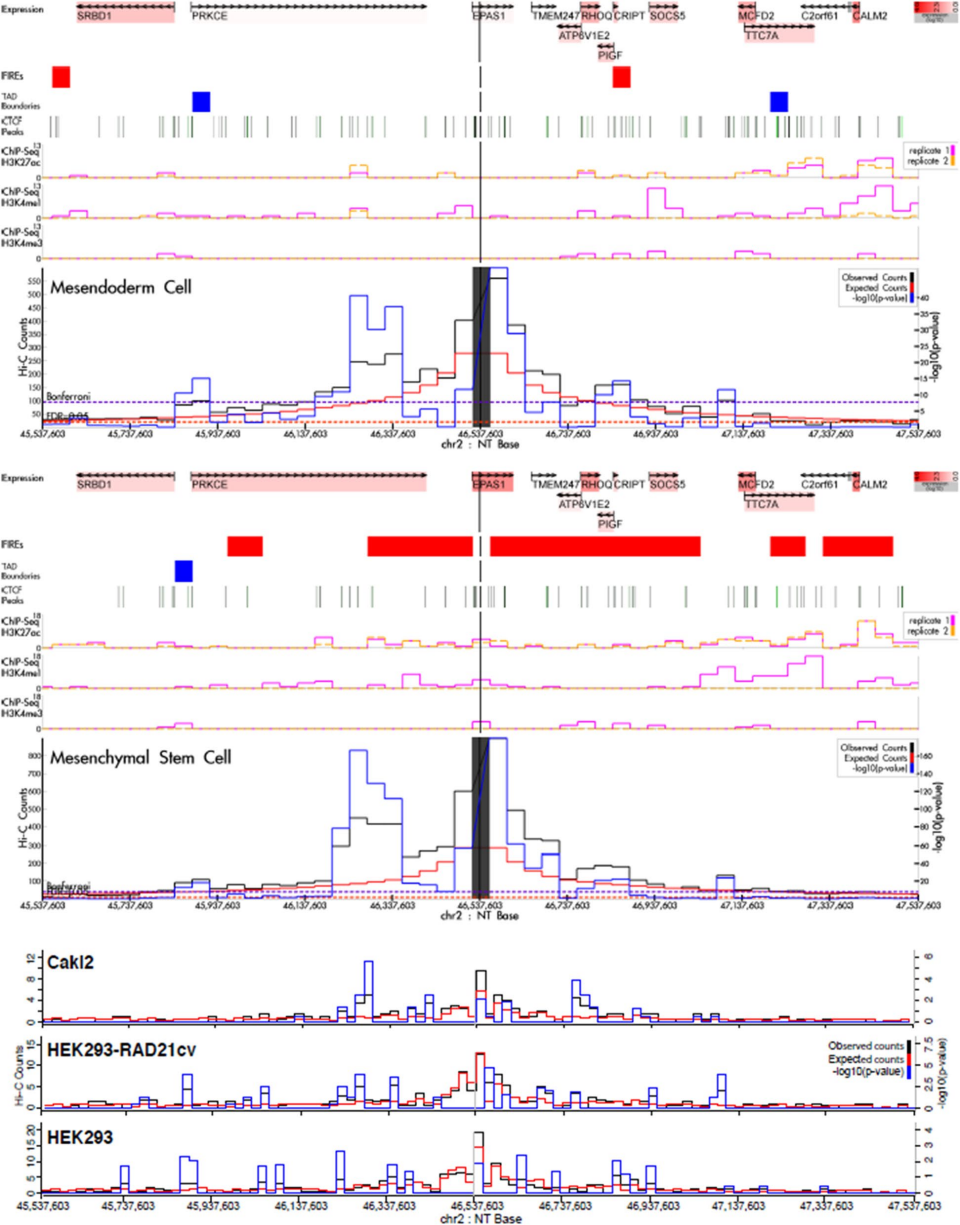
Hi-C interactions for the *EPAS1* germline variant rs7579899. This variant was found to interact with *EPAS1* and *PRKCE*

**Fig. 2:**
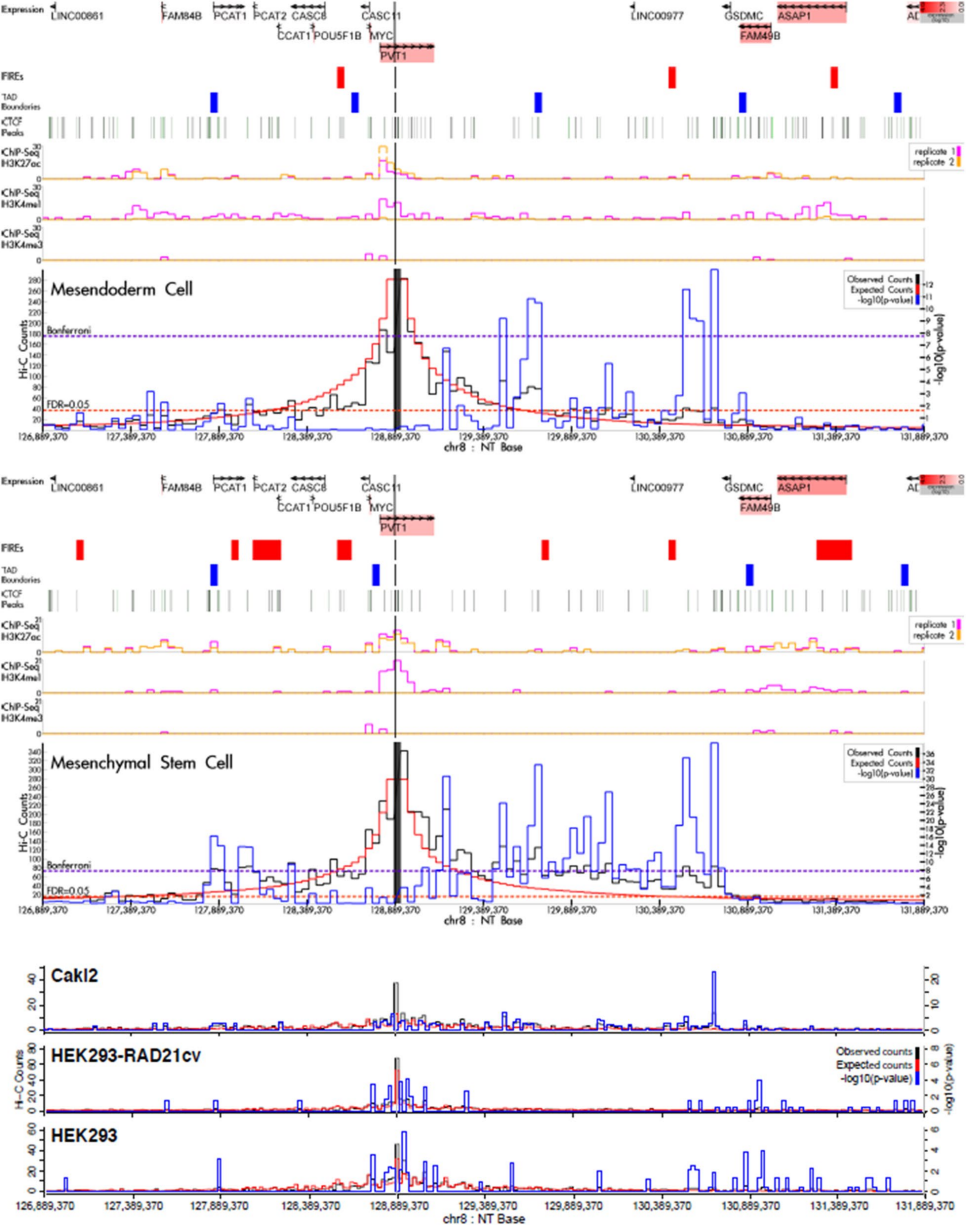
Hi-C interactions for the 8q24 germline variant rs35252396. This variant showed interactions with *PCAT1* and *PCAT2*

We evaluated the interaction of each of the known germline variants with putative target genes in mesendoderm cell lines and mesenchymal stem cells using publically-available Hi-C data. Hi-C identifies chromatin interactions to evaluate the three dimensional chromatin structures inside the nucleus, which may identify long-range interactions. Some germline variants demonstrated interactions with nearby genes: e.g., rs4381241 with *FAF1* (518.5 kb away), rs57579899 with *EPAS1* (16.8 kb away), rs12105918 with *ZEB2*/*ZEB2-AS1* (~ 70 kb away), rs1800057 with *ATM* (50.2 kb away) and rs4903064 with *DPF3* (81.4 kb away) (Fig. 1; Additional file 1: Figure S1). However, some of these variants showed additional long-range interactions that have not been reported previously: e.g., rs4381241 with *CDKN2C* and *TTC39A*, rs7579899 with *PRKCE*, rs12105918 with *ARHGAP15*, *GTDC1* and *TEX41*, rs1800057 with *CUL5*, *ACAT1*, *NPAT* and *EXPH5* and rs4903064 with *RGS6*. Other germline variants also demonstrated long-range interactions that have not been reported previously: e.g., rs10936602 with *SEC62* and *PHC3*, rs67311347 with *ENTPD3*, *CTNNB1* and *ULK4*, rs2241261 with *PEBP4* and *EGR3*, rs74911261 with *EXPH5*, rs718314 with *SSPN* and *ITPR2* and rs4765623 with *FAM101A* (Additional file 1: Figure S1). Additionally, rs35252396 demonstrated interactions with *PCAT1* and *PCAT2* in mesenchymal stem cells but not in mesendoderm cells (Fig. 2). Similarly, rs11813268 had interactions with *OBFC1* in mesenchymal stem cells but not in mesendoderm cells. Finally, we failed to identify genes within ± 1 Mb whose promoters interacted with rs7105934 in either mesenchymal stem cells or mesendoderm cells. All identified interactions were further evaluated in independent cell lines: *VHL*-mutant ccRCC cell line (Caki) and *VHL* wild-type embryonic kidney cell lines (HEK293 and HEK293 RAD21cv). A large proportion of the interactions were also identified in ccRCC or embryonic kidney cell lines. For example, rs10936602 interaction with *SEC62*, rs2241261 interactions with *PEBP4* and *EGR3*, rs1800057 and rs74911261 interactions with *EXPH5*, as well as rs718314 interaction with *SSPN* were confirmed in all three cell lines. Further, rs12105918 interaction with *GTDC1*, rs11813268 interaction with *OBFC1* and rs4765623 interaction with *FAM101A* were only identified in the ccRCC cell line, while rs7579899 interaction with *PRKCE*, rs35252396 interaction with *PCAT2*, and rs4903064 interaction with *DPF3* were only identified in the embryonic kidney cell lines.

## Discussion

There are currently 14 known germline variants that are associated with risk of RCC; however, it remains unclear how germline genetics modify the risk of developing RCC or their association with tumor aggressiveness. To date, associations between germlines genetics and tumor genetics is largely unknown for RCC. With respect to tumor aggressiveness, previous investigators did not observe significant associations between a polygenic risk model that was derived from these 14 germline variants and age at onset or tumor stage [5]. However, the analyses were performed on overall RCC and not within relevant RCC subtypes. Herein, we not only evaluated ccRCC specifically, but also the association of these 14 variants with specific molecular (*BAP1*, *PBRM1*, *SETD2* and *VHL*) and clinically-aggressive subtypes of ccRCC. Using a case-case analysis, we observed that the 8q24 germline variant rs35252396 was significantly associated with tumor *VHL* mutation status as well as with the Mayo SSIGN score. Notably, the Mayo SSIGN score has been reproducibly shown to be associated with clinical outcome [13–16]. The 8q24 germline variant is located within *PVT1*, a candidate oncogene that is thought to regulate *MYC* to promote tumor formation. Of note, the 8q24 variant also overlaps a DNase I hypersensitive site and H3K4me1 peak from fetal kidney [23], indicating its location within a regulatory region. Through functional laboratory studies, investigators recently demonstrated that the 8q24 germline variant affects *HIF* binding to a *MYC* enhancer [24]. While the *EPAS1* (rs7579899) and *CCND1* (rs7105934) variants are also linked to the *HIF* pathway, we did not observe a statistically-significant association between these two variants and *VHL* mutation. Additionally, while it did not pass our multiple testing significance threshold, we also observed a candidate association between the *EPAS1* germline variant rs57579899 and *SETD2* tumor mutation (*p* = 0.012). We previously reported that loss of *SETD2* activity was associated with greater risk of ccRCC death [25].

While previous investigators did not observe a significant association between a RCC-derived polygenic risk model and tumor stage [5], we observed a significant association between the 8q24 germline variant and the Mayo SSIGN score. The difference could be due to the fact that the original analysis [5] was performed for overall RCC whereas our analyses were performed within a more homogeneous subtype of RCC, particularly, ccRCC. The Mayo SSIGN score is derived from an additive model that contains tumor stage, tumor size, tumor grade and presence of necrosis and the model has reproducibly been shown to be associated with outcome in ccRCC, with higher SSIGN score being associated with poorer prognosis [13–16]. While we observed a significant association of the 8q24 germline variant and the Mayo SSIGN score, we did not observe a significant association between any of the 14 variants and the molecularly-defined ccA/ccB expression subtype that has been linked to ccRCC aggressiveness [11, 12, 17].

Previous studies have performed expression quantitative trait loci (eQTL) analyses to evaluate function of the 14 RCC germline variants [2, 5]. Herein, we used Hi-C data to identify candidate target genes underlying the association of each of the 14 germline variants with ccRCC risk. While some variants demonstrated interactions with nearby genes, we observed additional long-range interactions. Laboratory studies are necessary to further understand these observations.

This study has limitations. We only analyzed associations of germline variants with somatic mutations, and no other acquired molecular alterations such as copy number variation. Additionally, because there are limited GWAS data available on patients who also have tumor molecular data, we did not validate the observed associations between the 8q24 germline variant and *VHL* tumor mutation nor the association between the *EPAS1* germline variant and *SETD2* tumor mutation. Similarly, Hi-C data are currently limited and thus we were not able to validate the Hi-C results. As such, the observed associations reported herein require validation in an external cohort.

## Conclusion

We identified a significant association between the 8q24 germline variant and the presence of *VHL* somatic mutation. Additionally, we demonstrated for the first time an association between the 8q24 germline variant and ccRCC clinical aggressiveness as measured by the Mayo SSIGN score. Importantly, we additionally defined candidate target genes underlying the association between each of the 14 germline variants and risk of ccRCC. Together, these results further elucidate genes and pathways associated with ccRCC development. Specifically, these data further demonstrate the link between rs35252396 in the 8q24 region, *HIF* pathway and clinical aggressiveness, providing a more comprehensive biological understanding of the development of *VHL* mutated ccRCC tumors. Future work should evaluate how the rs35252396 germline variant and *VHL* mutation interact to affect treatment outcome and prognosis.

## Supplementary information

**Additional file 1: Supplementary Figure S1**. Hi-C interactions for each of the 14 known RCC germline variants. Chromosome 1: rs4381241. **Supplementary Table S1**. Information on the 4 known RCC germline variants. R-square denotes the imputation quality and FRQ denotes the frequency of the variants for each of the aquired alterations that were evaluated. **Supplementary Table S2**. Association of known RCC germline variants with age of diagnosis.

## Data Availability

The data that support the findings of this study are available from TCGA but restrictions apply to the availability of these data, which were used under license for the current study, and so are not publicly available. Data are however available from the authors upon reasonable request and with permission of TCGA.

## Abbreviations

ccA: Clear cell A
ccB: Clear cell B
ccRCC: Clear cell renal cell carcinoma
eQTL: Expression quantitative trait loci
FIREs: Frequently interacting regions
GWAS: Genome wide association study
kb: Kilobase
MAF: Minor allele frequency
OR: Odds ratio
*p*: *p* Value
RCC: Renal cell carcinoma
SSIGN: Stage, sign, grade and necrosis
TAD: Topologically associating domain
TCGA: The Cancer Genome Atlas
