# Supplementary material for: 8q24 clear cell renal cell carcinoma germline variant is associated with VHL mutation status and clinical aggressiveness
Source: BMC Urol. 2020 Oct 29;20:173. doi: 10.1186/s12894-020-00745-9 (PMC7597051; doi:10.1186/s12894-020-00745-9)

**Supplementary materials**

**8q24 Clear Cell Renal Cell Carcinoma Germline Variant is Associated with *VHL* Mutation Status and Clinical Aggressiveness**

Jeanette E. Eckel-Passow^1,*^, Huihuang Yan^1^, Matthew L. Kosel^1^, Daniel Serie^2^, Paul A. Decker^1^, Robert B. Jenkins^3^, Brian Costello^4^, Bradley Leibovich^4^, Thai H. Ho^5^, Alexander Parker^2^

**Supplementary Figure S1:** Hi-C interactions for each of the 14 known RCC germline variants.

Chromosome 1: rs4381241


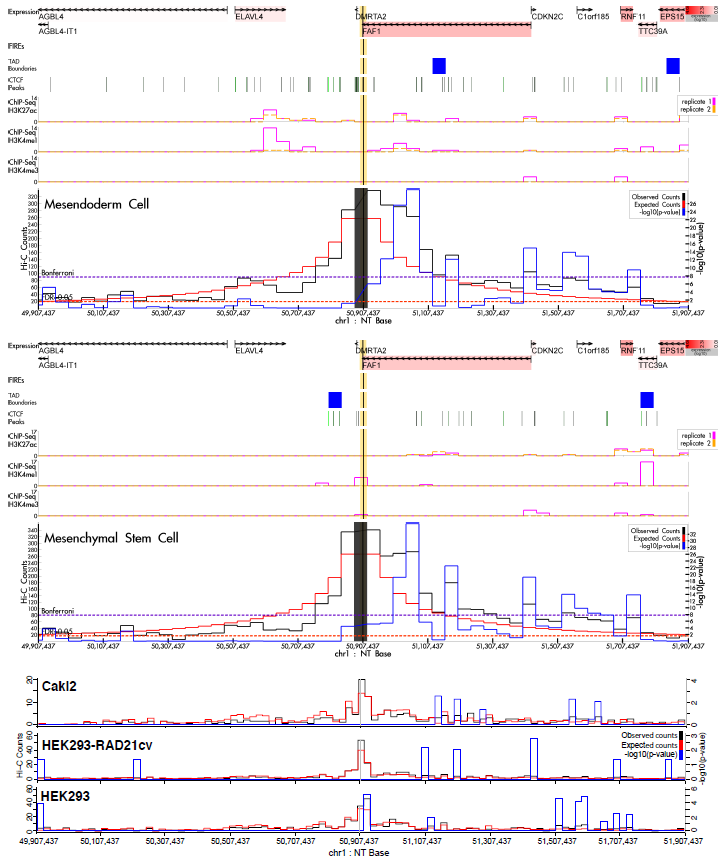


Chromosome 2: rs7579899


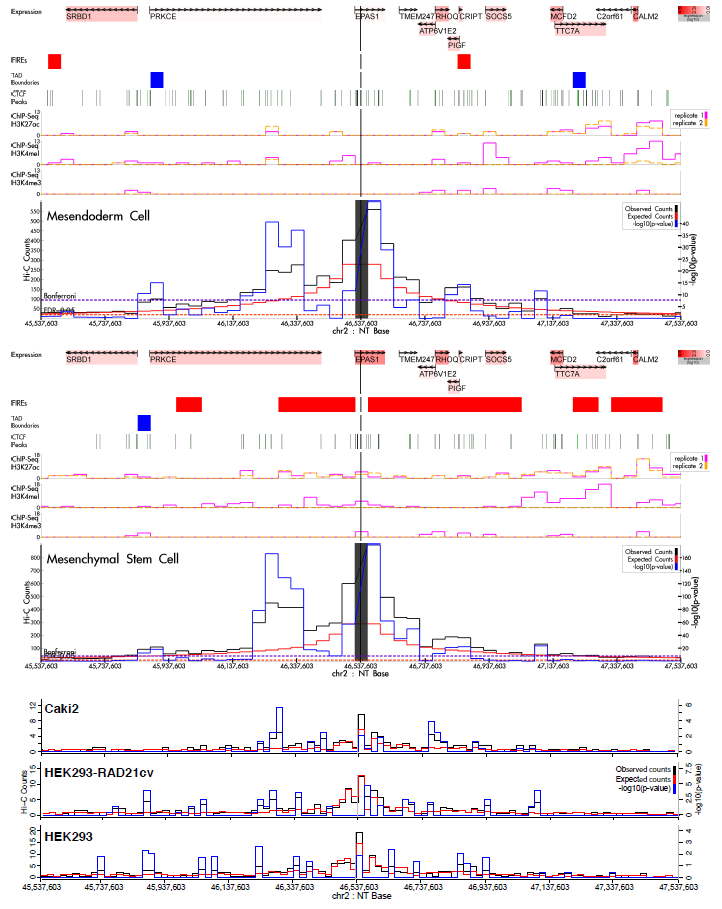


Chromosome 2: rs12105918


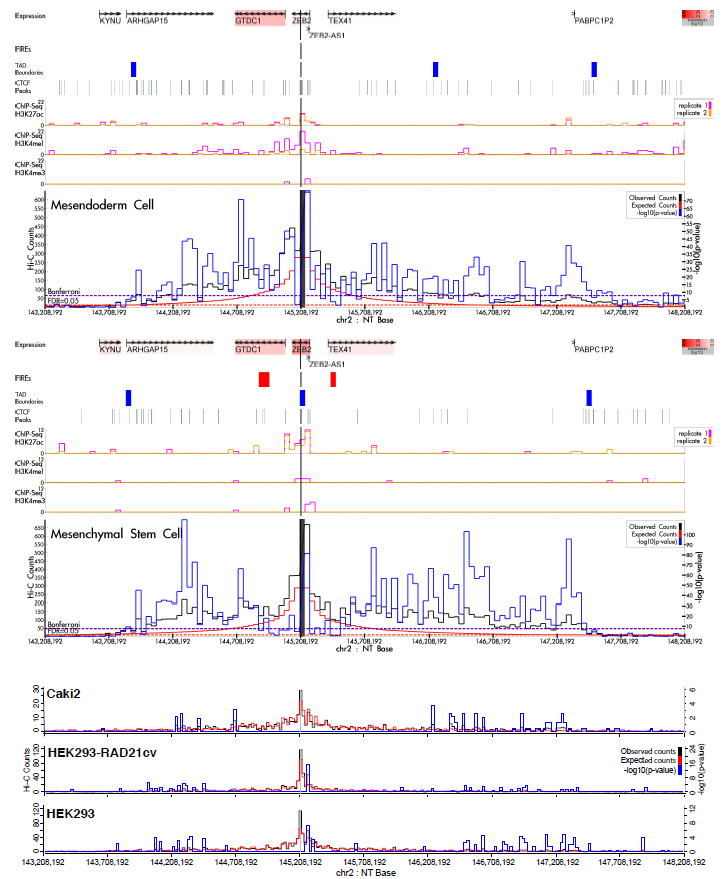


Chromosome 3: rs10936602


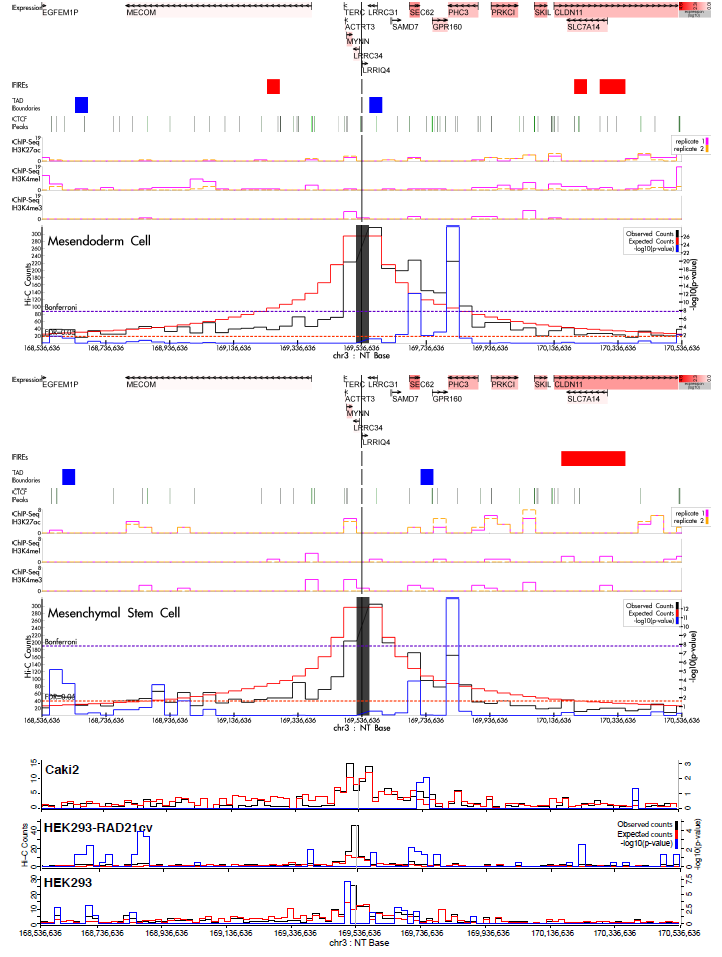


Chromosome 3: rs67311347


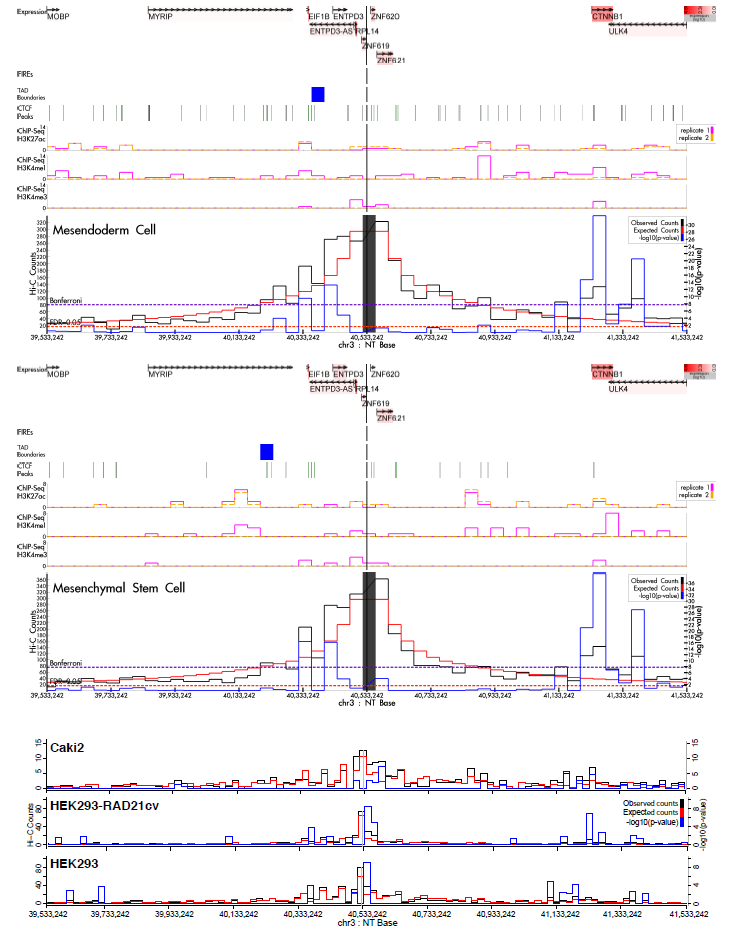


Chromosome 8: rs2241261


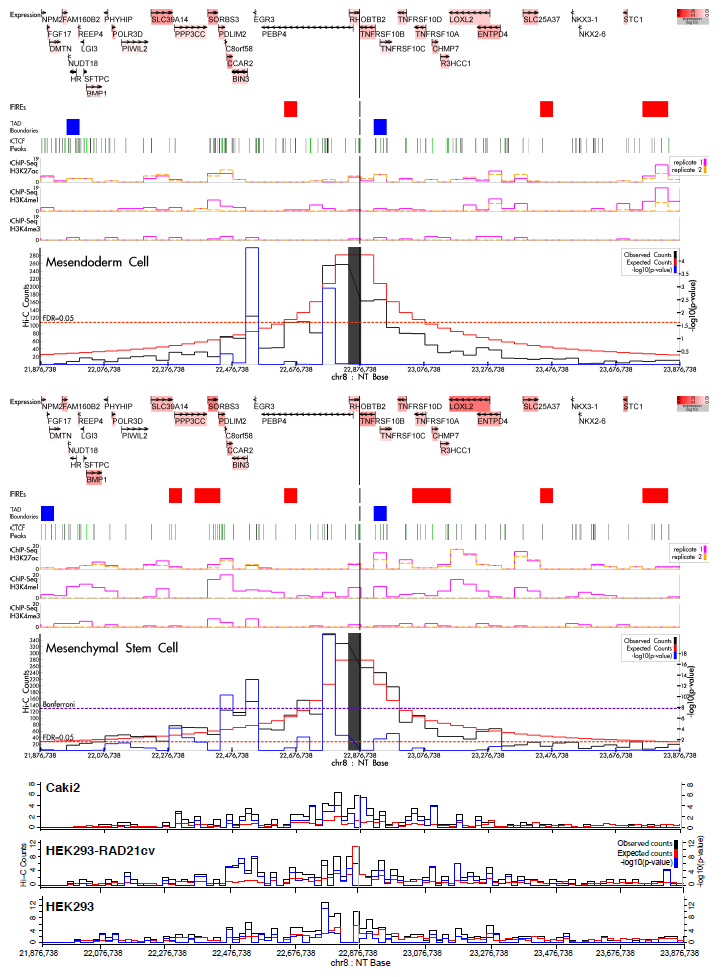


Chromosome 8: rs35252396


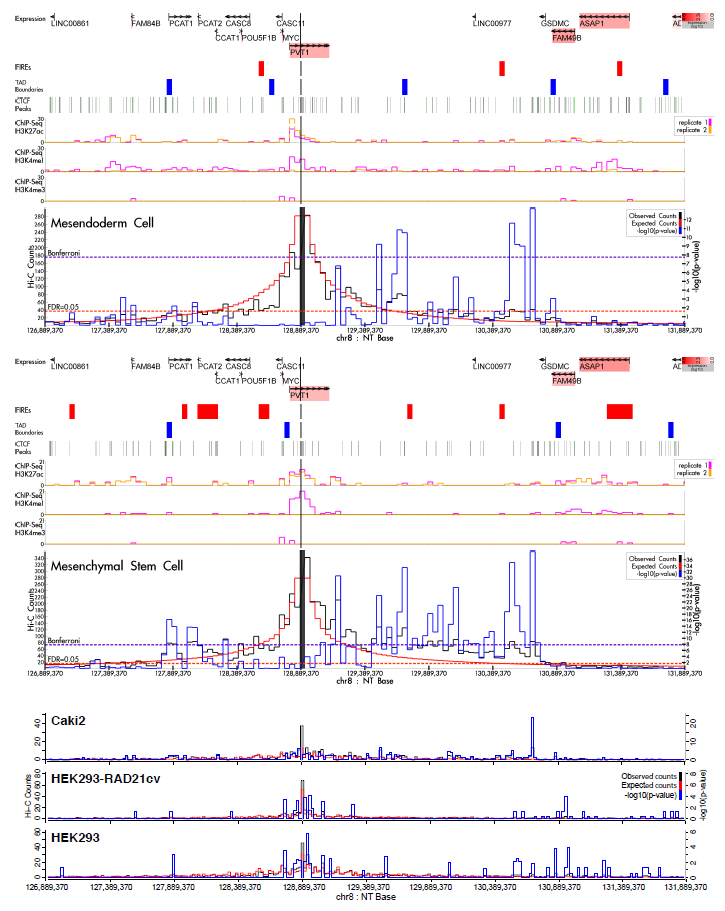


Chromosome 10: rs11813268


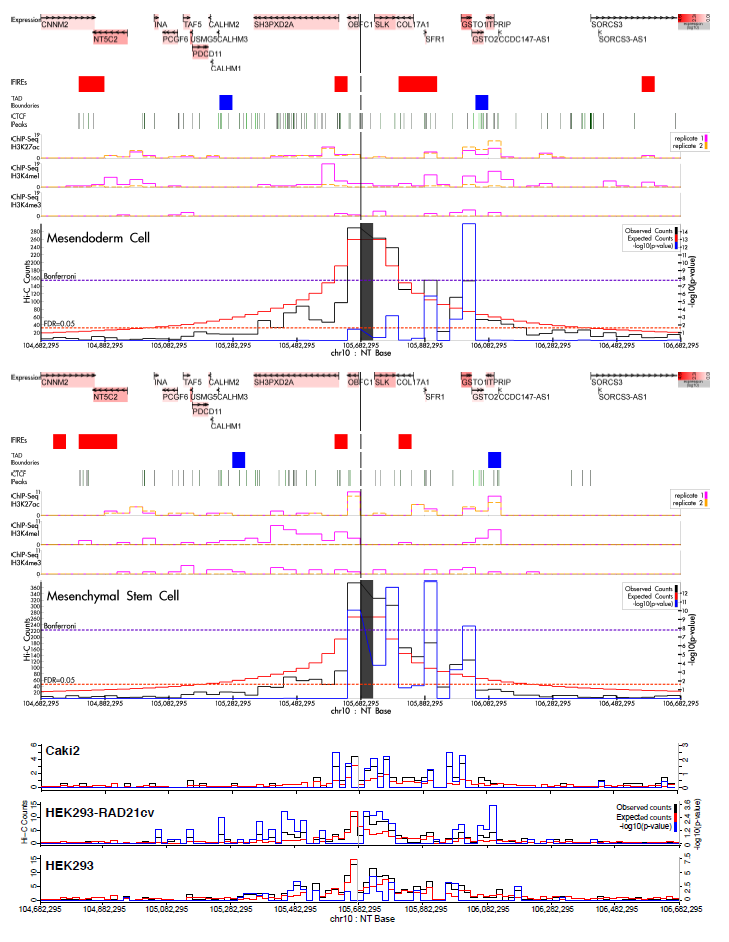


Chromosome 11: rs1800057


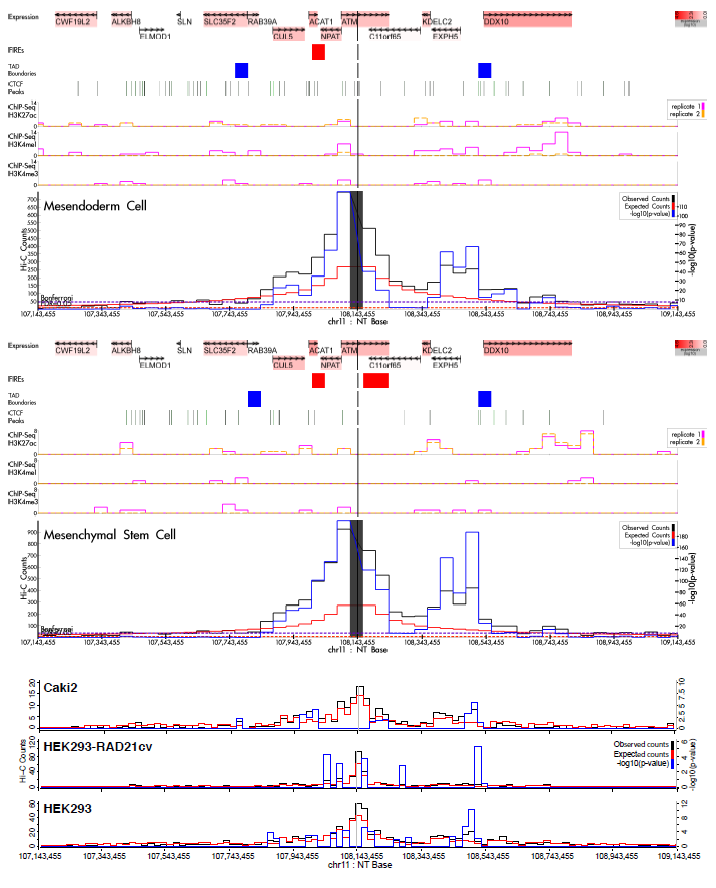


Chromosome 11: rs7105934


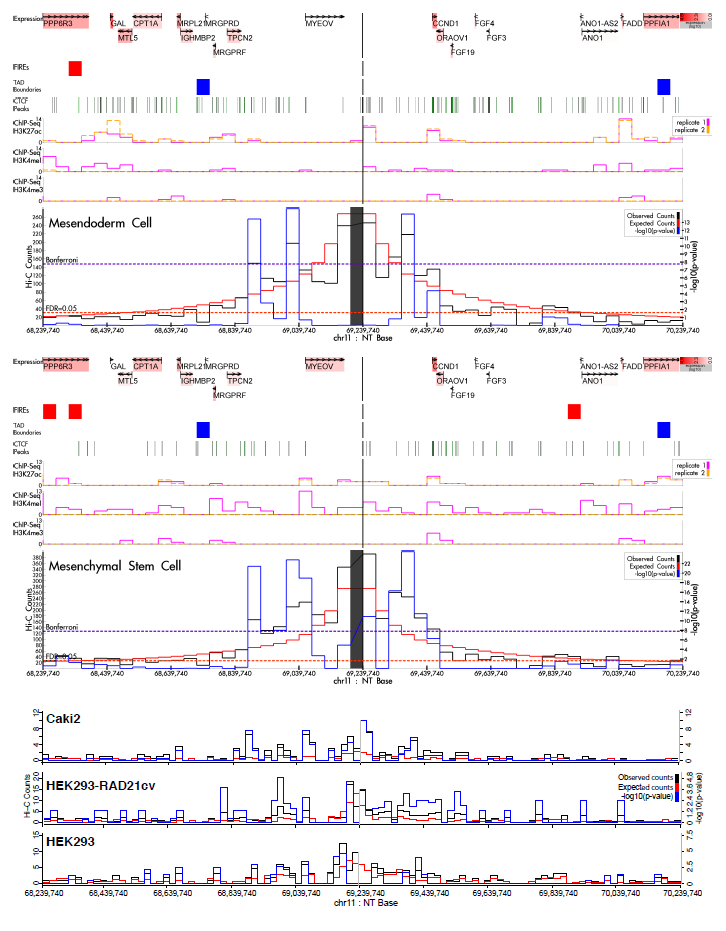


Chromosome 11: rs74911261


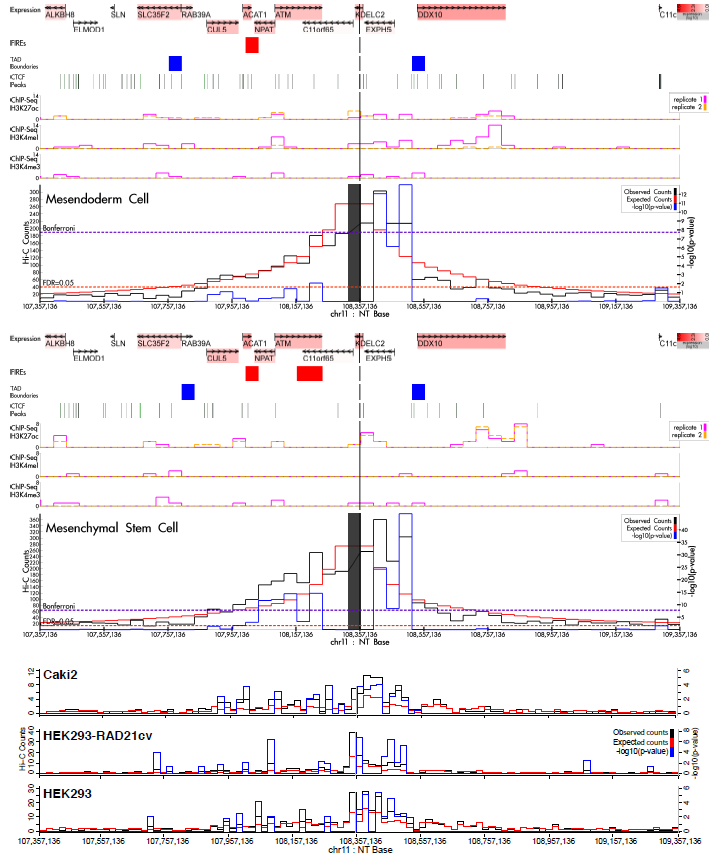


Chromosome 12: rs718314


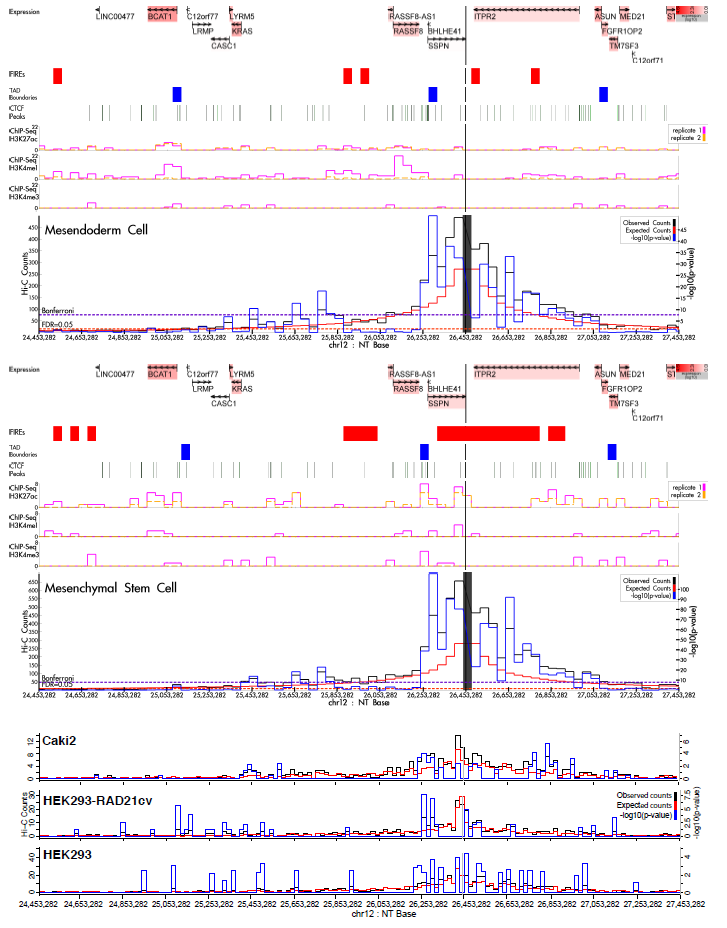


Chromosome 12: rs4765623


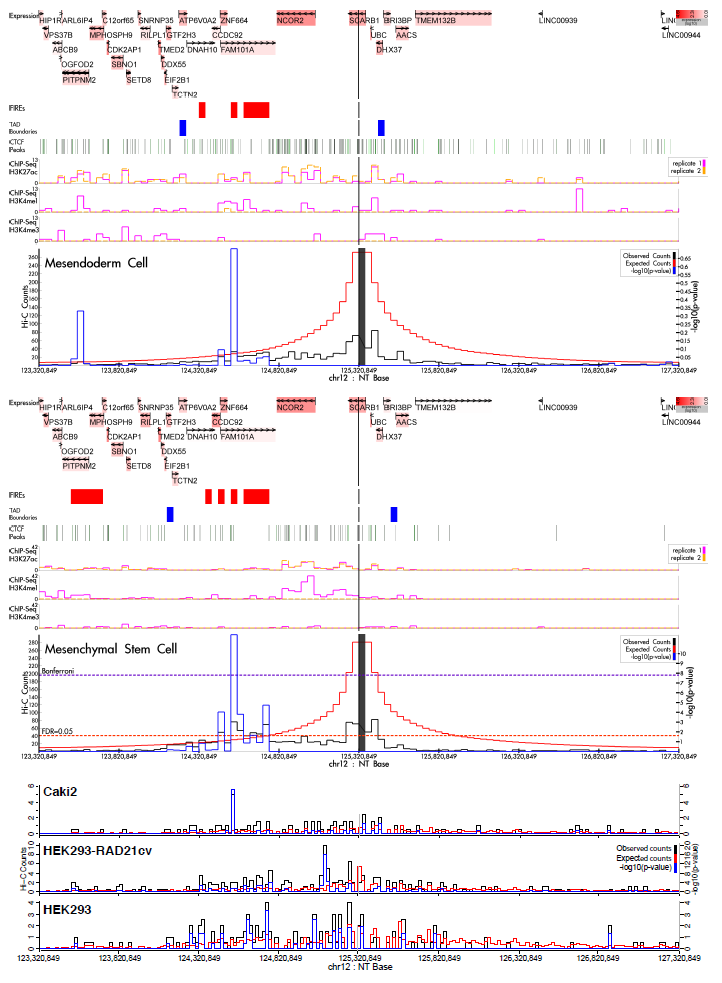


Chromosome 14: rs4903064


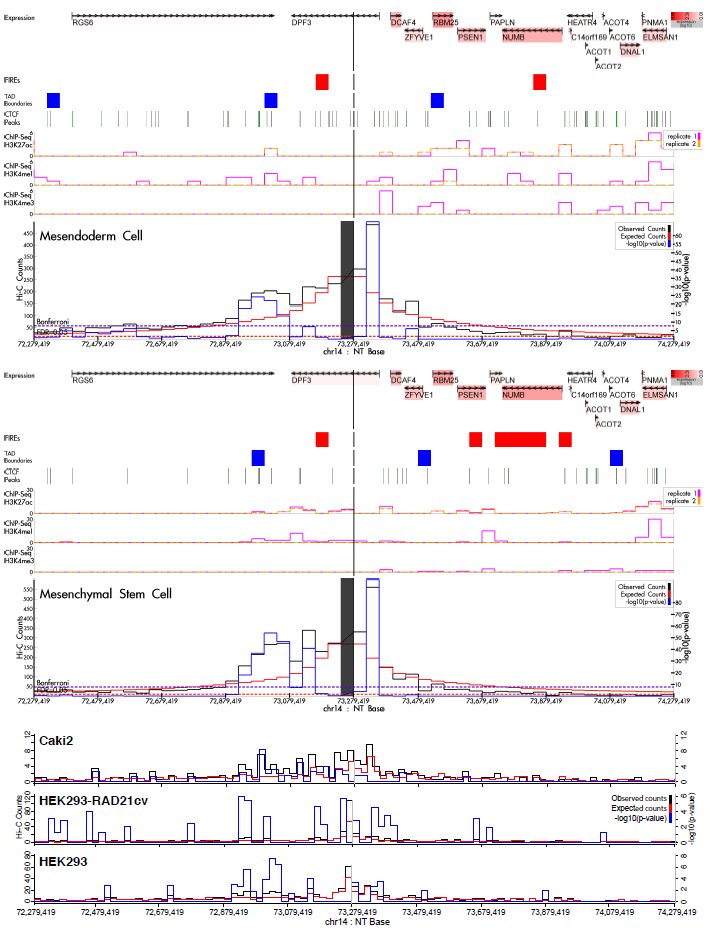

Supplement: Supplementary file 1 — Additional file 1: Supplementary Figure S1. Hi-C interactions for each of the 14 known RCC germline variants. Chromosome 1: rs4381241. Supplementary Table S1. Information on the 4 known RCC germline variants. R-square denotes the imputation quality and FRQ denotes the frequency of the variants for each of the aquired alterations that were evaluated. Supplementary Table S2. Association of known RCC germline variants with age of diagnosis. [file 12894_2020_745_MOESM1_ESM.docx]
